# Changes in the Oral Cavity in Menopausal Women—A Narrative Review

**DOI:** 10.3390/ijerph19010253

**Published:** 2021-12-27

**Authors:** Aleksandra Ciesielska, Aida Kusiak, Agata Ossowska, Magdalena Emilia Grzybowska

**Affiliations:** 1 Department of Periodontology and Oral Mucosa Diseases, Medical University of Gdańsk, 80-204 Gdansk, Poland; akusiak@gumed.edu.pl (A.K.); agata.ossa@wp.pl (A.O.); 2 Department of Gynecology, Gynecological Oncology and Gynecological Endocrinology, Medical University of Gdańsk, 80-204 Gdansk, Poland; magdalena.grzybowska@gumed.edu.pl

**Keywords:** menopausal women, menopause, oral health, saliva, oral mucosa, periodontitis

## Abstract

Oral health awareness during the menopausal period is essential to minimize the inevitable inconveniences which may occur due to hormonal changes. The decrease in estrogen hormone concentration impacts the oral mucosa in a similar way to the vaginal mucosa due to the presence of estrogen receptors in both of these structures. An estrogen deficiency also affects the maturation process of the oral mucosal epithelium and can lead to its thinning and atrophy, making it more susceptible to local mechanical injuries, causing a change in pain tolerance and problems in the use of removable prosthetic restorations. Mucosal epithelium during the menopausal period is more vulnerable to infections, *candidiasis*, burning mouth syndrome, oral lichen planus (OLP), or idiopathic neuropathy. Moreover, salivary glands are also hormone-dependent which leads to changes in saliva secretion and its consistency. In consequence, it may affect teeth and periodontal tissues, resulting in an increased risk of caries and periodontal disease in menopausal women. Due to the large variety of complaints and symptoms occurring in the oral cavity, menopausal women constitute a significant group of patients who should receive special preventive and therapeutic care from doctors and dentists in this particular period.

## 1. Introduction

Menopause is an inevitable condition that is part of every woman’s life. Most of them spend a third of their lives in this particular state after their reproductive years have ended. The World Health Organization defines this term as the last menstrual bleeding, which usually occurs between the ages of 45 and 55. This spontaneous cessation of natural menstruation for at least 12 months is not a pathological condition [1,2,3,4]. Although the influence of a decrease in estrogenic hormone concentration due to the discontinued endocrinological activity of the ovaries results in a number of systemic processes occurring in the women’s body causing the undesirable symptoms reported by women during this period [2,5,6]. As a result of the cessation of the protective effect of estrogen hormones on the blood vessel wall, ailments, such as hot flushes, night sweats, lipid disorders, and an increased risk of coronary artery disease appear. Additionally, they experience not only physical but also psychological ailments, such as sleep disturbance, irritability, anxiety, and depression [1,2,3,7]. The type of symptom group manifested the most may vary by race and ethnicity, as well as by factors, such as education level, socioeconomic status, health factors, stress, and marital status [8,9,10]. For example, African American women report significantly more vasomotor symptoms than Caucasian women, who experience more psychosomatic symptoms than other racial/ethnic groups [11]. A significant correlation between particularly higher psychosomatic symptoms and the lower socioeconomic status of women, regardless of age, race, menopause, and menopausal hormone therapy use is also observed [12]. Nevertheless, according to the literature, in the peri- and postmenopausal period, women develop a number of troublesome systemic symptoms, and among them, in particular, we can distinguish complaints from the oral cavity.

The oral cavity is a complex of many anatomical structures with different functions. It is made of both soft and hard tissues, and the scope of their activities extends very widely. The mucosa lining the oral cavity is very sensitive to both mechanical and chemical stimuli, therefore, the ailments located in this area are often very unpleasant and burdensome for patients. According to some studies, approximately 43% of postmenopausal women suffer from oral discomfort [13]. The most commonly reported symptoms are dryness, a sensation of decreased salivation, burning sensation of the oral mucosa, dysgeusia, altered pain tolerance and decreased tolerance to the use of removable prosthetic restorations [5]. Oral mucosa ailments often have a huge impact on the emotional state of suffering patients. Further worsening of unpleasant oral conditions accompanying menopause is associated with activation of the autonomic nervous system caused by chronic emotional anxiety. The general symptoms that occur in women during menopause are well documented. However, unfortunately, the level of awareness of the fact that oral discomfort in patients at this time can also be the result of hormonal changes is still low. Oral health awareness during the menopausal period is essential to minimize the inevitable inconveniences which may occur due to hormonal changes. The decrease in estrogen hormone concentration impacts the oral mucosa similarly like on the vaginal mucosa due to the presence of estrogen receptors in both of these structures [2,14,15,16,17]. An estrogen deficiency also affects the maturation process of the oral mucosal epithelium. It can lead to its thinning and atrophy, making it more susceptible to local mechanical injuries, causing a change in pain tolerance and problems in using removable prosthetic restorations [2,6,14,15,18]. Mucosal epithelium during the menopausal period is more vulnerable to infections, *Candidiasis*, burning mouth syndrome, oral lichen planus (OLP), or idiopathic neuropathy [18]. Moreover, salivary glands are also hormone-dependent, which leads to changes in saliva secretion and its consistency. In consequence, it may affect teeth and periodontal tissues, resulting in an increased risk of caries and periodontal disease [2,14,16,17,19,20,21,22].

Our review presents a number of the most frequently occurred changes in the oral cavity in menopausal women observed in our own study, with particular emphasis on the physicochemical and component changes in saliva, which, as a connecting medium of extraordinary importance for oral health, in the long-term perspective, may affect various structures of the oral cavity, inducing certain pathological changes directly or indirectly. It also draws attention to an equally important dimension of changes in the mental sphere of women in this period, which also can translate into changes occurring in the oral cavity and the intensity of perceived ailments. Moreover, it provides a number of general prophylactic and therapeutic recommendations for women that can help them avoid or overcome persistent oral discomfort often experienced during this particular period of their life.

## 2. Saliva of Menopausal Women

Dryness of the mouth or xerostomia is one of the most common symptoms reported by women in the menopausal period [2,14,15,16,17]. Xerostomia is a subjective feeling of dryness, most often caused by decreased salivation or when the salivary factor is normal, but with a reduced amount of its components. This was confirmed by a study in a group of women aged 52–73, conducted by Agha Hosseini et al. The stimulated saliva index was examined in 21 women with a feeling of dryness and 21 controls while assessing the composition of saliva [23]. Xerostomia most often affects patients over 50 years old and is particularly common in peri and postmenopausal women. These reports were confirmed in the studies of Asplund and Aberg, where in the age group of women 40–64 years, dryness before menopause was noted in 17.8% of the respondents, and in 23.3% of women during and 5 years after menopause. The results for 5–9 years and over 10 years after menopause were 29.2% and 34.5%, respectively [19]. Postmenopausal women compared with premenopausal women have decreased unstimulated and stimulated submandibular and sublingual salivary gland flow unrelated to any medication effect [24]. In this context, although an association to menopause exists, the decreased salivary flow is known to be related to age, though not always necessary. Although, of course, the additional presence of concomitant systemic diseases in menopausal women, such as diabetes, rheumatoid arthritis, Sjogrean’s syndrome, and the constant use of many medications, the side effect of which is xerostomia, increases the feeling of dry mouth [2,19]. This is confirmed by the studies by Ben Aryeh et al., whose aim was to compare the general symptoms of menopause and the condition of the oral cavity; 154 menopausal women participated in this study. Of these, 58 were generally healthy and were not taking any medications, and the remaining 94 were taking medication for various systemic illnesses. Discomfort and dry mouth occurred in 45% of healthy menopausal women and in 60% of those with systemic diseases [5]. Evaluation of salivary function in postmenopausal women to explain the association between dry mouth and menopause has been attempted in a number of studies. In the literature, frequent occurrence of dry mouth in the peri and postmenopausal periods is reported. An essential role in the etiology is presumed to be played by the physiological reduction of serum estrogen levels. The literature confirms the proven presence of estrogen receptors in both the oral mucosa and the salivary glands. Steroid hormones can also be assessed in saliva samples and their salivary concentrations correlate to those in serum. Salivary flow rates depend upon the estrogen status of the individual [2,14,15,16,17,25]. Many studies confirm slightly decreased salivation in menopausal women compared to women in the reproductive stage. Mahesh et al., Rukmini et al., Kullander et al., and Foglio-Bonda et al. obtained similar results, presenting a significant decrease in salivary flow rate during the menopausal period. Reduced saliva secretion, which is the principal defense factor in the oral cavity, can lead to a number of problems, such as the higher occurrence of dental caries, oral infections, dysphagia, taste disturbance, and increased sensitivity of the mucosa to mechanical injuries. A normal flow of unstimulated and stimulated saliva is important to ensure sufficient and continuous lubrication of the oral tissues. The fluid characteristics of saliva are essentials for food bolus formation, dissolving taste substances and transporting them to taste receptors. It also facilitates chewing, swallowing food and speech. Insufficient saliva can result in the disruption of the microbial balance to the benefit of pathogens, such as *Candida albicans* and *Streptococcus mutans* because the moist environment is also important for colonization and growth of microorganisms on oral surfaces [2,14,26,27,28,29]. However, some studies do not show a significant reduction in salivary flow rate in women during the menopausal period. Studies by Ship et al. and Ben Aryeh did not find any changes in this parameter [5,30]. A similar result was obtained by Agha-Hosseini et al. where no statistical difference was also found in the magnitude of the total stimulated saliva secretion coefficient between the study and control groups. However, it was found that in the group of menopausal women with reported dry mouth, the mean concentration of estrogen hormones was significantly lower [23]. Many researchers also emphasize the influence of the mental state of patients on the proper perception of the level of hydration of the mucosa. The sympathetic nervous system is involved in controlling the salivary glands. Saliva production is stimulated by the nerve branches of the autonomic system that regulate mucin secretion [2,14].

Furthermore, worth noting is the fact that during the menopausal period not only the amount of saliva secreted may change, but also its composition. The occurring changes in the level of steroid hormones affect the intestinal absorption of calcium into the body. Decreased concentration of estrogens, which occurs in the menopausal period, reduces intestinal calcium absorption. It leads to an increase in serum parathyroid hormone levels (PTH). PTH is a hormone that is responsible for the regulation of calcium and phosphate metabolism in the body. Disturbances in calcium-phosphate metabolism regulation result in increased calcium release into blood serum and saliva [14,21,22]. Studies provided by Agha-Hosseini et al., Sewon et al., and Cydejko et al. showed similar results, confirming the higher concentration of calcium in the saliva of menopausal women compared to the controls [22,23,31]. Therefore, a higher concentration of calcium in the saliva may affect the faster mineralization of plaque and increase calculus formation. These consequence factors may result in a greater risk of developing gingivitis and periodontitis. Several studies have shown that subjects with increased inorganic salivary calcium, pH, flow rate, and poor oral hygiene are at a higher risk of developing periodontitis as calcification of plaque occurs more readily in such patients [20,21,32,33]. On the other hand, the higher level of this parameter may increase the resistance to caries due to the better remineralization potential of saliva in these individuals [20,21,34]. Cydejko et al. also observed a statistically significant difference in the lysozyme concentration in the saliva of menopausal women compared to the control group. This parameter was significantly reduced compared to menstruating women [31]. Lysozyme, a part of the innate salivary defense mechanisms, presents antibacterial, antiviral, and antifungal activity. It occurs in saliva and gingival crevicular fluid. Its decreased level in menopausal women may affect the increased risk of oral candidiasis, periodontitis, and other oral infections [35,36,37,38,39,40]. However, due to the small amount of research in this area, broader research should be carried out on this topic. Considering the fact that oxidative stress in saliva plays an important role in the pathogenesis of oral diseases, Zovari et al. undertook studies to investigate the total antioxidant capacity and lipid peroxidation capacity of the serum and saliva of premenopausal and postmenopausal women. By determining the total antioxidant capacity (TAC) and malondialdehyde (MDA) values in saliva and serum, he confirmed the increase in oxidative stress in the serum of postmenopausal women, but no significant changes in the levels of MDA and TAC in saliva were found [41].

Recent studies confirm that xerostomia is also closely related to factors, such as the treatment of climacteric symptoms and some menopausal symptoms and has a profound impact on the quality of life of menopausal women [42]. The results achieved by Taga et al. suggest that oral sensory complaints (OSC), including xerostomia, taste disturbance, and burning mouth, are associated with the number of menopausal symptoms. Therefore, the management of general menopausal symptoms can reduce the incidence and severity of oral discomfort, improving the quality of life for women during this period [43]. Research by Davis et al. among Australian healthcare providers showed that although they appeared to have a solid understanding of menopause and its consequences, they were uncertain of its management. The commission of menopausal hormone therapy appeared to be limited to women with severe symptoms despite lifestyle changes and attempts to use complementary and alternative medicine [44]. Menopause hormone therapy (MHT), currently available in an ever-expanding array of formulations, routes of administration, and dosages, continues to appear to be the most effective treatment for menopause-related symptoms. However, the decision to implement it may be complex, especially in women with chronic medical conditions, such as diabetes, obesity and autoimmune diseases [45,46]. An attempt to evaluate the effectiveness of MHT in relieving oral symptoms in postmenopausal women with dry mouth was undertaken in a recent study by Wang et al. The study was conducted with the help of a questionnaire related to oral dryness and laboratory estimation of salivary estradiol levels in unstimulated saliva before and after case group was subjected to MHT. Oral dryness complaints were significantly reduced after the therapy and the mean salivary estradiol in the case group levels increased [47].

Table 1 included in our review summarizesthe main changes occurring in menopausal women in the parameters of saliva and other structures of the oral cavity that are directly and indirectly influenced by it. In Table 2, we have listed a series of practical prophylactic and therapeutic recommendations for menopausal women that can help them avoid or significantly reduce oral discomfort at this particular time in their lives.

## 3. Oral Mucosa in Menopausal Women

Changes in oral mucosa during menopause could be compared to changes in endometrium which correlate with estrogen deficiency. According to the research, the oral mucosa is similar to vaginal mucosa in its histology and they both contain estrogen receptors [14,15,16,17]. Hormonal changes have a strong influence on the women’s oral cavity in a direct way or indirectly by neural mechanisms. Atrophic changes in the oral mucosa can lead to the development of autoimmune lesions, such as pemphigus vulgaris, benign mucosal pemphigoid, oral lichen planus (OLP) and also other disorders like idiopathic neuropathy and increased tendency to *candidiasis* due to increased colonization of microorganisms [18,48,49]. Oral mucosa during the menopausal period becomes more prone to many infections [14,18]. The prevalence of oral lichen planus is higher in perimenopausal women (10.91%) than in premenopausal women (0.5–2.0%) and what is interesting it is more frequently observed in women with depression and psychological disorders. It is considered that declined levels of estrogen and progesterone can directly or indirectly (by causing the depression) provoke OLP [59]. Reduced salivation and dryness is the most common complaint reported by women after menopause. Minicucci et al.’s research confirmed that dry mouth is not present in all menopausal women, but the saliva flow is significantly reduced and its consistency is changed. This reduction may be due to the abnormalities or blocking of salivary gland function which the aging process and hormonal changes may cause. Nevertheless, hyposalivation is more frequently observed in menopausal women than in premenopausal women [48]. Therefore, in menopausal women, we are dealing with reduced hydration of the mucosa and thus depriving it of its natural protection provided by saliva under normal conditions, resulting from changes in its quantity and composition. Deprived of moisture, the mucosa becomes thin, atrophic, crimped, less flexible, and more easily susceptible to mechanical injuries and oral ulcerations following mechanical trauma due to abnormal oral habits or removable dentures [14,18]. Due to atrophic mucosa, dentures should be fabricated as smooth as possible to avoid additional traumatization of the oral mucosa. Furthermore, menopausal women are more prone to eating and gastric disorders. Recent reports suggest that the menopausal transition may represent a window of vulnerability for eating disorders in women [60]. Psychological changes and often observed depression may lead to self-induced vomiting and it may also lead to changes in the oral cavity like angular cheilitis, erythema, dehydration, trauma to the oral mucous membrane and pharynx [61].

Moreover, one of the most common symptoms of the oral mucosa reported by women during this period in the absence of clinically manifested infection is burning in the mouth. Burning mouth syndrome, also known as glossodynia, stomatodynia, stomatopyrosis, glossopyrosis or glossalgia is one of the most frequent oral symptoms during menopause, affecting women in the fourth or fifth decade of life. The disorder shows a clear female predominance (7/1 over males) and association with age. What is characteristic of burning mouth syndrome (BMS) is that there are no clinical lesions in oral mucosa, no laboratory abnormalities, or causative systemic disease but patients experience continuous burning pain of the mouth. The prevalence of oral discomfort was found to be significantly higher in perimenopausal and postmenopausal women (43%) than in premenopausal women (6%) [6,48,62]. The most common localization of the pain is the tongue, but other parts of the mouth like lips, palate, gums, or areas of denture support may be affected. Pain begins to appear 3 to 12 years after the onset of menopause. Moreover, the pain may be accompanied by oral dryness, hypersensitivity to some food compounds, alterations in taste (dysgeusia) and breath, swallowing difficulties and facial or dental pain [50,63,64]. The etiology of BMS is complex and multifactorial. The causes may be somatoform pain disorder, *candidiasis*, factors, such as personality, stress, anxiety, depression and other psychological, psychiatric disorders, or even genetic polymorphisms. Other systemic factors may be diabetes mellitus, B group vitamin deficiency (vitamins B1, B2, B6 and B12), folic acid and iron deficiency, hormonal imbalance, gastrointestinal diseases, and drug-induced side effects. Potential local causative factors may be mechanical irritation, parafunctions and dysfunctions of the stomatognathic system, contact allergy to dental materials and electro-galvanic phenomena [50,63,65]. Hormonal changes like lowering the level of estrogen and progesterone during the perimenopause period can affect the condition of the psyche [66]. Certain micro-organisms, such as Candida albicans, Staphylococci, Streptococci and various anaerobes have also been suggested as the etiological factor of burning mouth syndrome [67]. Jeong-Hyun Kang et al. has investigated oral mucosal epithelial membrane-bound mucin (MUC1) expression in women aged 60 ± 5 years and concluded that the disturbance in psychoendocrinological mechanisms might affect oral mucosal MUC1 expression and severity of oral burning feeling in post-menopausal BMS women. Salivary progesterone and 17β-estradiol levels had positive correlations with oral mucosal epithelial MUC1 expression level. However, salivary cortisol level had a negative interaction with the extent of oral burning sensation [49,68].

## 4. Periodontal Disease in Menopausal Women

Periodontal disease is an inflammatory process, which develops over time, ultimately leading to tooth loss. Its occurrence is strongly related to the accumulation of biofilm (dental plaque), bacterial dysbiosis, the formation of periodontal pockets, and tissue destruction, which includes alveolar bone loss [69]. According to the literature, it is a disease that may also affect menopausal women more often and present more severe form. The decrease in the estrogen level, typical in menopausal age, has a negative effect on the health of periodontium. Quantitative changes in sex steroid hormones can lead to changes in inflammatory mediators, vascular permeability, and the growth and differentiation of fibroblasts. Estrogen receptors are also present in cells of periodontal tissues, such as osteoblasts and fibroblasts. In consequence, during different stages of a woman’s life, different levels of hormones can influence changes in periodontal health status. It may cause the gums to become more susceptible to plaque and create a much higher risk for gingivitis and advanced periodontitis [6,51,52,53]. The influence of menopause on vascular inflammation and systemic bone loss has been documented. The Scardina and Messina study showed significant differences between post-menopausal and pre-menopausal women for the vascular parameters, such as the diameter of loops, tortuosity of vessels in labial mucosa, and density of periodontal mucosa. Their results confirm that tissues in the study group can be more susceptible to inflammation than controls [64]. A study provided by Baltacıoğlu et al. also suggests that menopause may be a risk factor for periodontitis. Their study established a relationship between menopause and periodontitis based on the association of oxidative stress and decreased antioxidant defense (AO) in these women. Comparing the total antioxidant capacity (TAOC) and the concentration of superoxide dismutase (SOD) in the serum and gingival crevicular fluid (GCF), they observed a decrease in systemic and local AO defense due to menopause and periodontitis with the lowest AO values in the group of postmenopausal women [70]. Moreover, the role of sex hormone levels and hormone replacement therapy in the occurrence of subgingival bacterial infections in peri and postmenopausal women is of great interest. Studies have already found a higher incidence of periodontitis in postmenopausal women who have not received menopausal hormone therapy (MHT) than in premenopausal women [71]. Additionally, it has been pointed out that changes in the state of the periodontium are associated with changes in the level of sex hormones. Research conducted by López-Marcos et al. confirmed the improvement of the parameters of the probing depth of periodontal pockets and the reduction of tooth mobility in women who received hormone therapy for 1 year [72]. Similarly, a cohort study by Tarkkila et al. showed that menopausal hormone therapy reduced the number of positive samples showing the periodontal pathogens *Porphyromonas gingivalis*, *Prevotella intermedia*, and *Tannerella forsythia* in the subgingival plaque [73]. However, not all studies confirm this correlation. Pilgram et al., when examining a group of women receiving estrogen therapy for 3 years, did not find any changes in clinical parameters [74]. Therefore, the scope of research on this subject should definitely be expanded even further.

Many studies also seem to show a correlation between the increased incidence of osteoporosis in postmenopausal women and periodontal disease. Estrogen deficiency plays an important role in the pathogenesis of postmenopausal osteoporosis; this is mainly due to the recognition that estrogen regulates bone remodeling by modulating the production of cytokines and growth factors from the bone marrow and bone cells. It leads to the upregulation of immune cells (macrophages and monocytes) and osteoclasts, which are responsible for the greater production of bone-resorbing cytokines [54]. Both of these diseases, osteoporosis and periodontitis, are characterized by bone resorption. Osteoporosis is a systemic bone resorption disease while periodontal disease involves local inflammatory bone loss, following an infectious breach of the alveolar bone, and it may result in tooth loss. Alveolar osteoporosis may increase the periodontal susceptibility to infection due to the weakened resistance of the bone tissue. The association of osteoporosis and periodontitis was confirmed in studies on radiographic measurements and also clinical parameters. Both diseases share risk factors, such as age, genetics, hormonal changes, and smoking, and can also be risk factors for each other. Due to their interaction, they also require simultaneous therapeutic management [55,56]. A certain correlation between systemic osteoporosis in postmenopausal women and increased alveolar bone loss in the course of periodontitis was confirmed by studies conducted by Pereira et al. and Bertulucci et al. [57,58]. A study by Mashalkar et al. involving 94 postmenopausal women aged 45–65 years showed that the level of osteoporosis is correlated with the level of periodontitis. After prior measurement of bone mineral density (BMD), all examined women were assessed as normal, osteopenic and osteoporotic on the basis of the obtained result and subjected to a detailed periodontal examination using clinical parameters, such as the oral hygiene index (OHI), the dental plaque index (PI), probing pocket depth and clinical attachment loss to check the severity of periodontitis [75]. Additionally, subsequent studies conducted by Zhu et al. in 2020 on a wider group of female participants (195 postmenopausal women aged 50–65 years) including periodontal examination and bone mass density (BMD) confirmed that postmenopausal women with osteoporosis have a greater chance of presenting severe periodontal attachment loss [76]. In addition, the resorption of the ridge after tooth extraction in postmenopausal women is also greater than in premenopausal women [24]. However, not all studies confirm the correlation between postmenopausal osteoporosis and the progression of parameters of periodontal disease. Ortman et al. showed no significant effect of menopause on bone resorption [77]. Imirzalioglu et al. only showed the influence of age on the degree of ridge resorption [78]. On the other hand, Sultan and Rao, in their studies of 80 postmenopausal women with chronic generalized periodontitis, obtained a result of an insignificant clinical correlation between attachment loss and alveolar bone loss with BMD [79]. Menopausal women diagnosed with osteoporosis, due to taking anti-resorptive drugs from the bisphosphonate group, also constitute a specific group of patients for dental treatment due to the risk of medication-related osteonecrosis of the jaw (MONJ), which may occur after procedures that violate continuity of bone tissue, most often after tooth extraction. The unique predisposition of the jawbones to the occurrence of osteonecrosis results from a high degree of bone transformation [80]. Any bone damage should be avoided during surgical treatment of patients taking bisphosphonates (BP). In some cases, the general implementation of antibiotic therapy before the planned procedure is required. In turn, in patients with periodontal disease, BP can be used to reduce alveolar bone loss. A number of published studies report that women who took oral bisphosphonates during menopause showed an improvement in periodontal status and a higher level of alveolar bone compared to the control group [81].

It should definitely be noted that the relationship between the physiological process of menopause in women and the risk of increased progression of periodontitis is complex due to the many factors involved. One of them can also be a psychological factor. Previous studies have assessed menopause as a risk factor for periodontitis, but due to a range of controversial research findings, to date, no consensus has been reached for a confirmed increased risk of periodontitis after menopause. However, the confirmed influence of emotional stress on the risk of periodontitis and the increased risk of depression in women undergoing the menopausal transition should also be taken into account. Longitudinal studies have demonstrated an association between the menopause transition and an increase in depressive symptoms [82]. Yoshida et al.’s study in a group of Japanese females between 40 and 59 years of age showed that depressive tendencies during menopause are more common among women with oral health problems [83]. There are several hypotheses to explain the association between depression and periodontal diseases. Earlier reports indicated that a depressed mood contributes to the occurrence of negligent oral hygiene. Increased dental plaque levels may lead to an inflammatory breakdown of the periodontal tissues, and vice-versa, systemic inflammation induced by the presence of periodontitis may lead to depression [84,85]. A study by Yakar et al. confirmed there is an association between the number of missing teeth, poor emotional well-being, and menopause [86]. However, due to the fact that both depression and periodontitis are chronic in nature, further research in this field should be carried out to assess their patterns of progression and mutual correlations during menopause.

## 5. Conclusions

Menopause is an inevitable period in every woman’s life, although unfortunately it is often neglected by healthcare providers. Oral lesions that appear at any stage of menopause are slightly less characteristic than systemic symptoms and are often non-specific but are noticeable. Their long-term effect is often persistent discomfort from the oral mucosa and changes appearing in the periodontium. Reduced salivation and changes in the composition of saliva can significantly affect the health of the oral mucosa, teeth and periodontium, and increase susceptibility to infections and mechanical injuries. Unfortunately, these aliments are often not associated with this particular period when estrogen hormone levels drop, leading to physiological changes in many tissues, including the oral cavity. Medical consultation is often an indispensable element, because the problem of menopause, although it is a natural physiological process, can often cause serious pathological changes. Therefore, due to the large variety of complaints and symptoms occurring in the oral cavity, menopausal women constitute a significant group of patients who should receive special preventive and therapeutic care from doctors and dentists in this particular period of their life. A detailed history of systemic diseases and the use of drugs should be underlined during the medical visit. A full clinical intraoral examination, complete evaluation of the mucosal membranes, examination of the periodontal and dental conditions, and salivary flow assessment should be conducted. Oral health is an integral part of general health and has a direct effect on the overall physical and mental health of an individual. As we can see, the early detection of oral changes in postmenopausal women is very important and should be considered along with other systemic changes. There is also a need for guidelines for menopausal women regarding good oral health and lifestyle.

## Figures and Tables

**Table 1:** Changes in the oral cavity of menopausal women in terms of parameters of saliva, mucosa, teeth and periodontium.

	Changes Occurring in the Oral Cavity of Menopausal Women
Saliva parameters	Decreased unstimulated and stimulated value of salivary flow rate [26,27,28] Increased concentration of inorganic salivary calcium [22,23,31] Reduced concentration of salivary lysozyme presenting antibacterial, antiviral, and antifungal activity [31]
Oral mucosa	Reduced hydration of the mucosa, making it thin, atrophic, folded, less elastic and more susceptible to mechanical injuries [14,18] Atrophic changes that can lead to the development of autoimmune changes, such as pemphigus vulgaris, benign mucosal pemphigoid, oral lichen planus, and other disorders, such as idiopathic neuropathy [18,48,49] Increased susceptibility to infections [14,18] Increased tendency to manifest symptoms of BMS [18,48,49,50]
Teeth and periodontium	Increased susceptibility to caries due to reduced salivation [2,14,26,27,28,29] Changes in inflammatory mediators, vascular permeability, and the growth and differentiation of periodontal fibroblasts leading to an increased risk of periodontitis [6,51,52,53] Faster mineralization of dental plaque and increased calculus formation due to a higher concentration of ionized calcium in saliva, which also affects the risk of periodontitis [20,21,32,33] Correlation between the increased risk of osteoporosis in women with estrogen deficiency and periodontitis [54,55,56,57,58]

**Table 2:** Prophylactic and treatment recommendations for women in the menopausal period.

Prophylactic and Treatment Recommendations for Women in the Menopausal Period
Initial examination of the oral cavity, including examination of the teeth, periodontium and special assessment of the mucosa in terms of its hydration and the presence of pathological changes, along with a possible sialometric test, which will allow determining individual treatment needs and possible indications for specialist treatment, such as periodontal or surgical treatment. Individualized instruction in oral hygiene, which is extremely important in the prevention of caries and periodontal diseases. Contraindicated use of mouthwashes containing alcohol and toothpaste with whitening agents and sodium lauryl sulfate, which increase dry mouth and irritate the oral mucosa. Recommendation of a mild diet that eliminates food with hard texture that may more easily injure the sensitive mucosa, spicy foods, sweet carbonated drinks and alcohol. Recommendation of local application to the oral mucosa of natural moisturizing and coating preparations, such as linseed oil, evening primrose oil, linseed, mallow flower, which have a soothing effect and protect against traumatic external factors. The use of artificial saliva preparations, e.g., mucin-based substitutes or preparations based on carboxymethylcellulose. Scheduled follow-up visits to the dentist, depending on the clinical situation, up to 3–6 months. In case of persistent ailments from the oral cavity that are not susceptible to local prophylactic and therapeutic procedures, it is advisable to order a gynecological consultation to implement possible menopausal hormone therapy (MHT).

## Data Availability

No new data were created or analyzed in this study. Data sharing is not applicable to this article.
